# Supplementary material for: Monilinia fructicola Response to White Light
Source: J Fungi (Basel). 2023 Oct 4;9(10):988. doi: 10.3390/jof9100988 (PMC10607740; doi:10.3390/jof9100988)

Supplementary Table S1. qPCR primers used in this study.

| Gene name | Primer ID | 5´-3´Sequence | Reference |
|---|---|---|---|
| Histone H3 | MlH3F | TCCGTCGTTACCAAAAGTCG | Rodríguez-Pires et al. (2020) |
| | MlH3R | GGCGAGTTGGATGTCCTTAG | |
| MFCVVD1 | Mfcvvd1F | ATTGCAGATTCCTACAACAGCC | This study |
| | Mfcvvd1R | GCTTGAAATCCGACCACATAAT | |
| MFCFAR1 | Mfcfar1F | GGCTCTGGTAGTACCTCCACCC | This study |
| | Mfcfar1R | TCCACATAGATCCCACAAACCC | |
| MFCCRY1 | Mfccry1F | CAATAGAGGAAGAGGTGGTGGAG | This study |
| | Mfccry1R | TTCCAGTTCGCAACTCCCTTC | |
| MFCCRY2 | Mcry2F | TGGGCTTTATTGATGCCTCG | This study |
| | Mcry2R | CCTGCAACATATTGCCAATTACC | |
| MFCPHY1 | Mphy1F | GGAGAAGAATGTGCAAGTGCCTA | This study |
| | Mphy1R | CGTTCGACAAGAGAGGCTGATAC | |
| MFCPHY2 | Mphy2F | ATGGTCAAGGGCGATGCTACT | This study |
| | Mphy2R | GGCCAACACCGAGAGTCTCTC | |
| MFCOP1 | Mop1F | TCTTCGCTGCTTACCACAACG | This study |
| | Mop1R | ACCTCAGAATCAACCGACCATG | |
| MFCOP2 | Mop2F | CTAGCCAAGATTGGCTTCGGT | This study |
| | Mop2R | ATCGTTGGCAGGATTATTCGAG | |
| MFCVEL2 | Mvel2F | CCAACTCATCGTGCTCCGTC | This study |
| | Mvel2R | CCTCGGCTACCATCCTTTCC | |
| MFCVEL4 | Mvel4F | TGCCGACTTGTCCGTTCG | This study |
| | Mvel4R | TTGGCTAACAAGAGATTTGGCC | |

Supplementary Table S2. Differentially expressed genes shared between samples from 5 minutes and 14h of illumination.

| Upregulated genes | | Downregulated genes | |
|---|---|---|---|
| Protein ID | Annotation | Protein ID | Annotation |
| MFRU_003g00560 | pnl1 | MFRU_003g05160 | hypothetical protein MFRU_003g05160 |
| MFRU_021g00620 | far1 | MFRU_028g00860 | Oxidoreductases |
| MFRU_008g01270 | HAD-superfamily class IIA hydrolase | MFRU_053g00330 | Oxidoreductases |
| MFRU_003g04280 | Unknown function | MFRU_036g00470 | putative exo-beta glucanase protein |
| MFRU_002g03330 | beta-sandwich domain of Sec23/24 | MFRU_006g02210 | Acting on a peroxide as acceptor |
| MFRU_071g00010 | Choline dehydrogenase | MFRU_028g00160 | putative epl1 protein |
| MFRU_005g02620 | SUR7 | MFRU_008g03590 | Translocases |
| MFRU_003g01680 | Cinnamyl alcohol dehydrogenases (CAD) | MFRU_032g00410 | putative cnd3 protein |
| MFRU_031g00840 | Taurine catabolism dioxygenase TauD | MFRU_050g00430 | putative multicopper oxidase protein |
| MFRU_005g04180 | Unknown function | | |
| MFRU_071g00130 | Unknown function | | |
| MFRU_014g01250 | Unknown function | | |
| MFRU_063g00080 | Taurine catabolism dioxygenase TauD | | |
| MFRU_004g03450 | Heat shock protein 9/12 | | |
| MFRU_008g00380 | Unknown function | | |
| MFRU_005g01380 | Major Facilitator Superfamily | | |
| MFRU_019g00940 | pg6 | | |
| MFRU_028g01080 | Lactonase, 7-bladed | | |
| MFRU_037g00110 | putative magnesium ion transporter protein | | |
| MFRU_003g02440 | cel1 | | |
| MFRU_013g00700 | Unknown function | | |
| MFRU_012g02510 | putative alkyl hydroperoxide reductase subunit c | | |
| MFRU_031g01060 | putative xenobiotic compound family protein | | |
| MFRU_009g02400 | putative zinc-binding dehydrogenase protein | | |
| MFRU_012g01220 | putative histidine acid phosphatase protein | | |
| MFRU_029g00280 | D-threo-aldose 1-dehydrogenase | | |
| MFRU_004g04290 | Unknown function | | |
| MFRU_002g03840 | Unknown function | | |
| MFRU_003g00720 | Transferring one-carbon groups | | |
| MFRU_024g00750 | putative integral membrane protein | | |
| MFRU_010g02220 | Oxidoreductases | | |
| MFRU_036g00130 | Acting on ester bonds | | |
| MFRU_019g01430 | putative major royal jelly protein | | |

Supplementary Figure S1. Light wave composition of used light sources

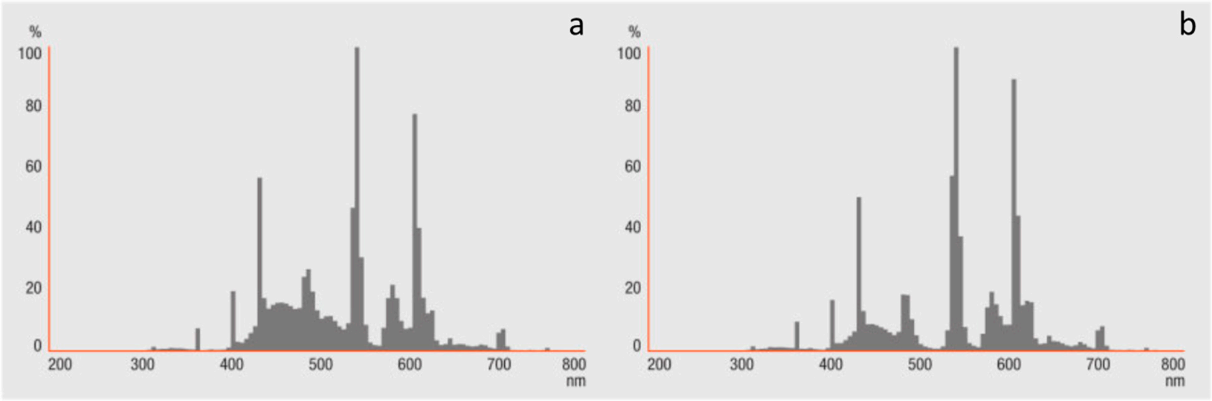


(a) Osram36W/954 “daylight”, (b) Osram 58W/840 “Cool White”.

Supplementary Figure S2. Relative abundance of species encoding a Far1-like protein within Pezizomycotina classes.

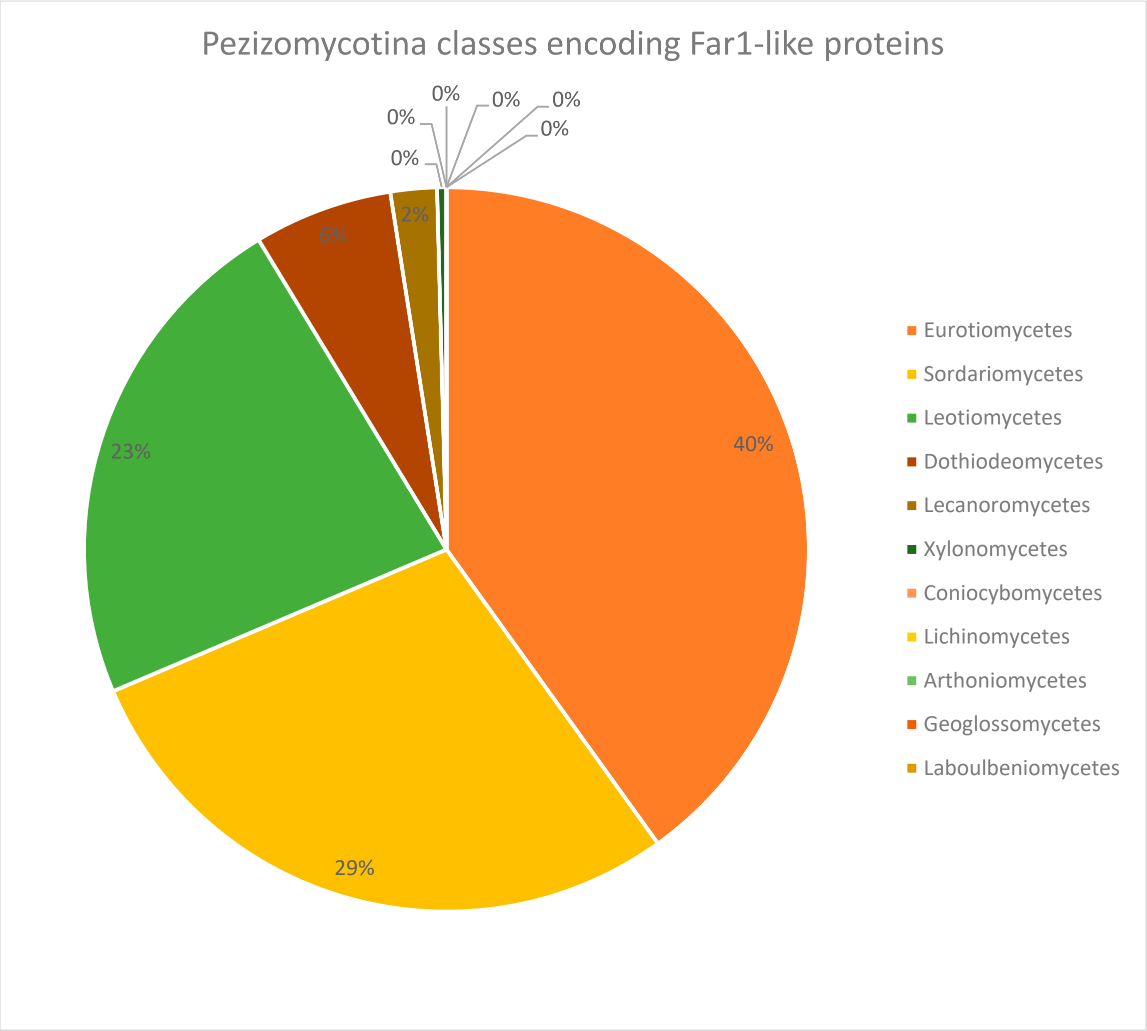

Supplement: Supplementary file 1 [file jof-09-00988-s001.zip › Supplementary Material.pdf]
